# Supplementary material for: SVR Chemometrics to Quantify β-Lactoglobulin and α-Lactalbumin in Milk Using MIR
Source: Foods. 2024 Jan 3;13(1):166. doi: 10.3390/foods13010166 (PMC10778881; doi:10.3390/foods13010166)
Supplement: Supplementary file 1 [file foods-13-00166-s001.zip › foods-2769120-supplementary.pdf]

Supplementary materials.

Outlier detection

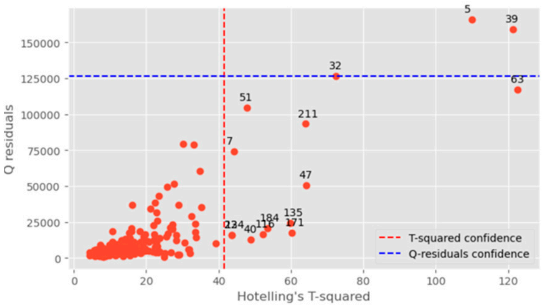

Figure S1. Outlier detection using Hotelling T2 and Q-residuals. The blue and red dashed lines are the 99% confidence interval for Q-residuals and Hotelling T2 respectively. The red circles represent sample replicates. Red circles with text are outliers with the text being the index of the replicate in the dataset. Samples that fall outside the either Q-residuals or Hotelling T2 confidence intervals are regarded as outliers.

Relationship between β-lactoglobulin and α-lactalbumin

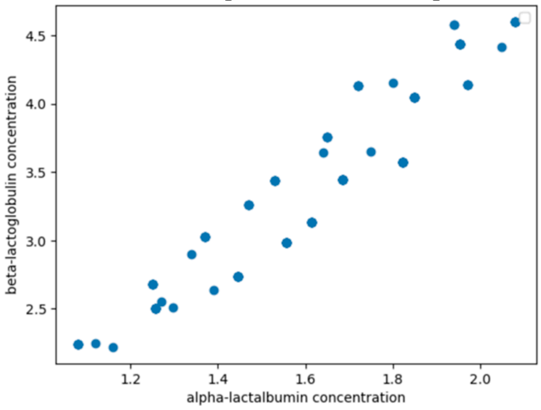

Figure S2. Relationship between β-lactoglobulin and α-lactalbumin

The plot in **Figure S2** shows a linear trend between β-lactoglobulin and α-lactalbumin concentration values.

Table S1. Preprocessing techniques applied and their parameter configurations.

Technique	Parameter(s)
**MC**	None
**SNV**	None
**MSC**	None
	Filter_window= 77, 99, 115, 151
**SavGol**	Poly_order = 1,2,3
	Deriv_order = 0,1
**EMSC**	None
**LSNV**	None
**RNV**	IQR= [90, 10], [75,25]
**Normalize**	None

MC-mean centering; SNV-standard normal variate; MSC-multiplicative scatter correction; SavGol-Savitzky Golay; EMSC-extended multiplicative scatter correction; LSNV-localize standard normal variate; RNV- Robust normal variate; IQR-interquartile range; n_comps-number of PLS components.

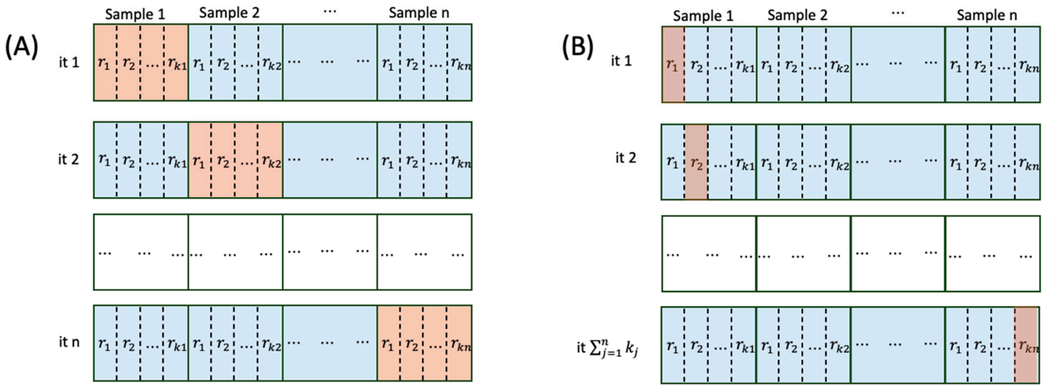

Figure S3. Schematic diagram of the LOOCV workflow for training and evaluating the performance of the chemometric models. (A) LOSOCV (B) LOROCV. The replicates are separated using the dashed lines and are represented as ri where i is the index of the replicate within the sample. Regions in orange color are the test samples while those in blue color are the training samples. The ith training iteration is represented as "it i".

Table S2. GA parameters and their corresponding values.

Parameter	Value
Fitness Function	R2 value of PLS model
Population Size	30
Selection Method	roulette wheel selection
Crossover Method	One-point crossover
Crossover Probability	50%
Mutation Method	bits-flip mutation
Mutation Probability	1%
Termination Criteria	100 runs
Maximum Number of Components	15

Table S2 gives the parameter used for the GA analysis. The fitness function assesses each chromosome's quality by maximizing its R2. The population size equals the user-defined number of chromosomes which is 30 in our case. Selection employs a roulette wheel mechanism, favoring chromosomes with high fitness values. Crossover probability is set at 50%, and mutation probability at 1%. The crossover determines the likelihood of two selected chromosomes undergoing crossover to create new offspring while the mutation determines the likelihood of individual bits (data points) within a chromosome undergoing mutation. The GA conducts 100 runs. PLS component selection occurs during the GA, using 15 components as the maximum n_components.

Table S3. Equidistant intervals comprising the starting datapoint, ending datapoint, and number of wavenumber data points in each interval.

Interval	Start wav.	End wav.	Number of data points
1	525.025	698.587	721
2	698.828	872.39	721
3	872.631	1046.19	721
4	1046.43	1220	721
5	1220.24	1393.8	721
6	1394.04	1567.6	721
7	1567.84	1741.4	721
8	1741.65	1915.21	721
9	1915.45	2089.01	721
10	2089.25	2262.81	721

11	2263.06	2436.62	721
12	2436.86	2610.42	721
13	2610.66	2784.22	721
14	2784.47	2958.03	721
15	2958.27	3131.83	721
16	3132.07	3305.63	721
17	3305.87	3479.2	720
18	3479.44	3652.76	720
19	3653	3826.32	720
20	3826.56	3999.88	720

Table S3 gives the wavenumber data points corresponding to each interval. With 14,416 data points, each interval initially contains approximately 720.8 data points, rounded up to 721, until we reach interval 17, where the number becomes evenly divisible by 720.

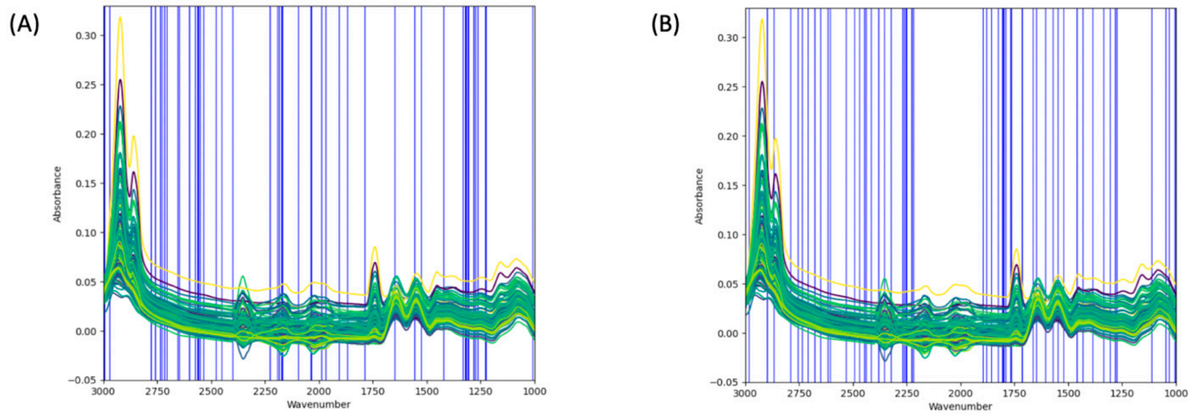

Figure S4. Selected wavenumbers data points for (A) β-LG and (B) -LA using simulated annealing. The areas in white background are the excluded wavenumbers and those in blue are the selected wavenumbers.

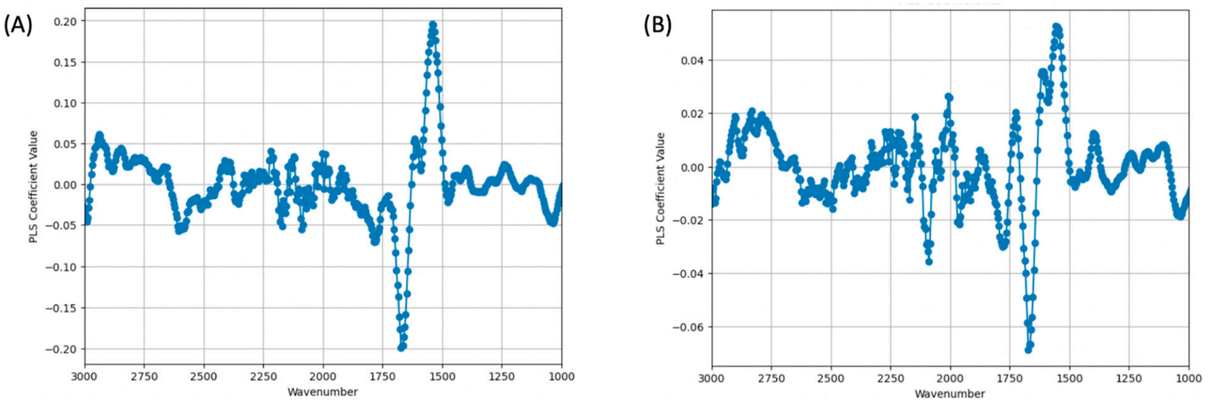

Figure S5. The magnitude and direction of wavenumbers selection for (A) β-LG and (B) -LA using PLS coefficient scores. Selected wavenumbers for (1)β-LG: 1502-1580, 1606-1618, 2914-2952 (2) α-LA:1518-1626, 1726, 2004-2015, 2790-2837 cm^{-1}.

The coefficient values of the wavenumbers data points are given in **Figure S5.** The data points with high coefficients, regardless of their directions (positive or negative) are the best performing data points.

Table S4. Interval discarded across different iterations using BiPLS and the model's performance after discarding the interval.

Iteration	Interval Discarded	RMSE
1	20	0.0915
2	16	0.0887
3	9	0.0871
4	11	0.086
5	7	0.0852
6	4	0.0848
7	3	0.0844
8	5	0.0841
9	2	0.0839
10	13	0.0839
11	15	0.0859
12	18	0.0863
13	8	0.0875
14	10	0.0895
15	12	0.092

16	19	0.0936
17	1	0.0942
18	17	0.0949
19	14	0.0961
20	6	0.0978

Table S4 gives the intervals discarded by BiPLS in each iteration along with their corresponding RMSE. The RMSE consistently decreased until iteration 10, after which it started to increase. This indicates that discarding intervals after iteration 9 did not improve RMSE further. Therefore, we selected the optimal intervals (13, 15, 18, 8, 10, 12, 19, 1, 17, 14, 6) corresponding to iterations 10 to 20. The wavenumbers corresponding to each interval are the same as those given in **Table S3.**

Table S5. Intervals selected as best performing intervals using SiPLS.

PLS comp.	Selected Intervals	RMSE
10	[1, 2, 6, 7]	0.0915
9	[1, 5, 6, 7]	0.0887
9	[1, 4, 6, 7]	0.0871
8	[1, 6, 7, 8]	0.086
9	[1, 6, 7, 13]	0.0852
8	[1, 3, 6, 7]	0.0848
10	[2, 4, 6, 7]	0.0844
9	[3, 4, 6, 7]	0.0841
10	[2, 3, 6, 7]	0.0839
10	[1, 6, 7, 11]	0.0839

The SiPLS results in **Table S5** display selected intervals and their corresponding RMSE values. SiPLS, an extension of iPLS, combines multiple intervals to build PLS models, and it becomes more computationally expensive as the number of interval combinations increases. The model's iterations are calculated as nCr, with higher n and r values leading to more iterations. In this study, we used 20 intervals for n and 4 interval combinations for r, resulting in the best intervals: [2, 3, 6, 7] and [1, 6, 7, 11], both with an RMSE of 0.0839. All selected intervals in Table S5 include intervals 6 and 7, corresponding to wavenumbers in the amide II and I regions.

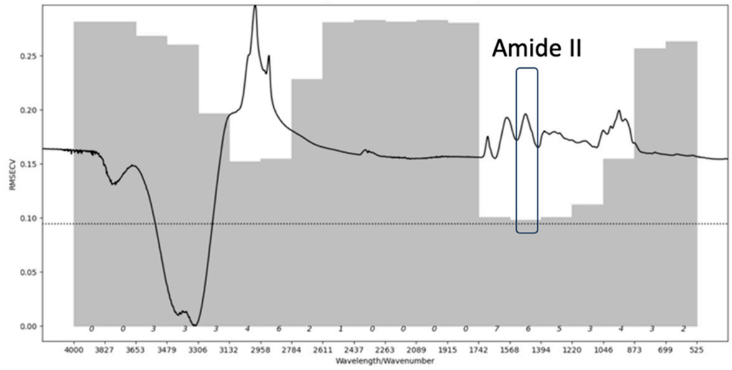

Figure S6. Optimal wavenumber selection using iPLS. The bars in gray indicate the RMSECV for each interval.

From **Figure S6**, we tested different numbers of equidistant intervals including 20, 25, and 30. However, 20 intervals were selected because it gave a clearer selection and evaluation of the different peaks. iPLS identified the Amide II region as the region with the least RMSECV. It could be observed that wavenumbers outside the range 873-3000 cm-1 appeared to be insignificant due to the high RMSECV values.

GA selection frequency results

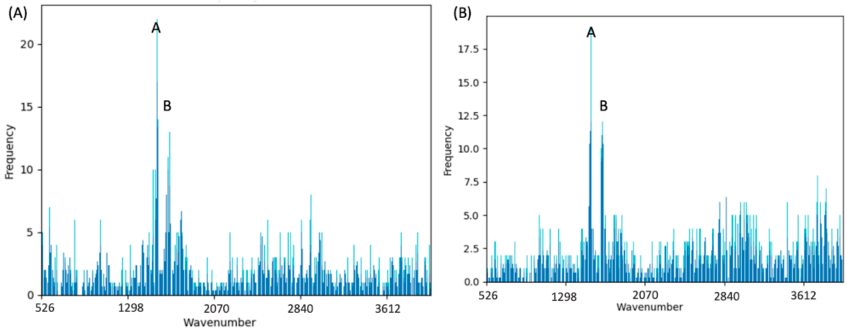

Figure S7. Wavenumber frequency selection for (A) β-LG and (B) α-LA.
A- Amide II region, B- Amide I region

Figure S7 shows that wavenumber datapoints in the amide I and II regions are the most frequently selected by the GA.
